# Myocardial Infarction after Endoscopic Removal of Foreign Body

**DOI:** 10.1155/2017/4541587

**Published:** 2017-02-26

**Authors:** Carola Maraboto, Florentino Lupercio, Ileana L. Piña

**Affiliations:** ^1^Department of Internal Medicine, Jacobi Medical Center/Montefiore Hospital and Medical Center, Bronx, NY, USA; ^2^Department of Cardiology, Montefiore Hospital and Medical Center, Bronx, NY, USA

## Abstract

The development of cardiac complications during or after endoscopic procedures is rare. However, mortality from myocardial ischemia, particularly in the elderly population, is elevated. We illustrate the rare case of a 79-year-old man with multiple cardiovascular risk factors who developed a non-ST elevation myocardial infarction (NSTEMI) after endoscopic removal of a foreign body. This case report summarizes a rare complication of a low-risk procedure and highlights the importance of considering this potential adverse event, particularly in patients with significant cardiovascular risk factors, to promote early diagnosis and proper treatment.

## 1. Introduction

Incidence of Acute Coronary Syndromes (ACS) following endoscopic procedures is low (<1%), and usually these interventions do not require further preoperative testing [1].

Cardiac complications are 2–5 times more likely to occur during emergency procedures than with elective interventions [2]. There are different tools to estimate risk of complications before an invasive procedure; one of the most widely used is the Revised Cardiac Risk Index, by Lee et al. [3], which can reliably predict postoperative cardiac outcomes and lead to interventions to lower preoperative risk; however this is usually not feasible in emergency situations.

## 2. Case Presentation

**Figure 1:**
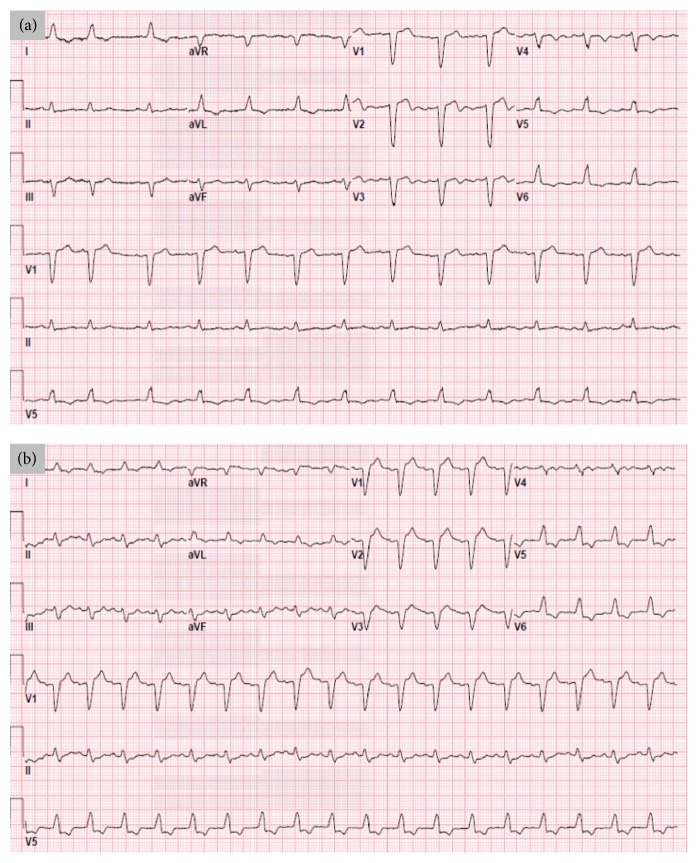
(a) Initial 12-lead ECG after removal of foreign body showing sinus rhythm, LBBB, and one supraventricular premature complex. (b) Second 12-lead ECG showing sinus tachycardia, the same LBBB, and new ST depressions in II, III, and aVF, as well as more prominent ST depression in V5 and V6 in the setting of LBBB.

A 79-year-old man, former smoker and with type 2 diabetes, hypertension, and hyperlipidemia, presented to the hospital after swallowing a piece of denture while eating carrots. On arrival physical exam and laboratory tests were unremarkable. Computed tomography (CT) scan showed esophageal distention compatible with ingested foreign body, as well as aortic and coronary atherosclerosis. A flexible esophagogastroduodenoscopy was done attempting foreign body removal; however it was unsuccessful and during the procedure ST-segment elevation was noted on the cardiac monitor. An emergent rigid esophagoscopy was performed and the foreign object was removed. Electrocardiogram (ECG) showed a left bundle branch block (LBBB) that did not meet Sgarbossa criteria; however there was no prior ECG to compare with (Figure 1(a)); vital signs were normal and initial troponin T was undetectable. Physical exam revealed chest crepitus and X-ray showed subcutaneous emphysema with pneumomediastinum, which was thought to be secondary to esophageal microperforations.

Repeat ECG (Figure 1(b)) showed sinus tachycardia, LBBB, and more prominent ST depressions in inferior and lateral leads; patient denied chest pain or dyspnea but reported mild abdominal pain. Troponin T rose to 4.72 ng/mL (reference range 0.00–0.10 ng/mL) and diagnosis of NSTEMI was made; management for ACS was started.

Coronary angiography was attempted once but patient became very agitated during the procedure; the Interventional Cardiology team increased the sedation to try to finish the study but patient remained restless and kept moving all his extremities, so continuing the procedure under these circumstances would have been extremely dangerous.

Transthoracic echocardiogram (TTE) revealed anteroseptum, apex, and distal-anterior wall akinesis; left ventricular ejection fraction was 30% (normal 5 years back). Troponin T peaked at 9.37 ng/mL and then trended down. Before being discharged, the medical team discussed extensively the importance of having a coronary arteriogram done to evaluate the presence of obstructive lesions in the coronary vasculature. Despite the insistence of the team and the broad discussion with the patient regarding different methods of anesthesia, patient refused to undergo this procedure given his prior experience. He remained hemodynamically stable and was discharged in stable condition.

## 3. Discussion

This is, to the best of our knowledge, the first case report of NSTEMI associated with esophageal microperforation after endoscopy in the setting of foreign body ingestion. A few reports do exist; however, of myocardial ischemia following gastroscopy [4], suggesting this procedure conveys an elevated risk in patients with underlying coronary artery disease.

Myocardial ischemia results from imbalance between oxygen demand and supply. During endoscopy, catecholamine-induced tachycardia can trigger it in patients with high atheromatous burden and fixed coronary stenosis or with development of hypoxia during a difficult procedure. In our patient, the ACS could have been induced by stress of aspirated dentures and the intervention triggering any of these mechanisms; however we were unable to exclude other etiologies such as Takotsubo cardiomyopathy.

Adverse cardiopulmonary events attributable to endoscopic procedures are rare [5–9] and range from hypoxia to pneumonia, respiratory arrest, ACS, stroke, and shock [10]. Nevertheless, mortality after acute myocardial infarction increases dramatically in the elderly population [11–14]. Cardiac complications result from a combination of factors specific to the patient, the procedure, and the surrounding circumstances. Patient-related factors include male gender, advanced age, cardiopulmonary disease, and increased Revised Cardiac Risk Index [15, 16]; procedure-related factors include technical difficulty causing hypoxemia [9, 17–19]. Our patient had significant cardiovascular risk factors, but no evidence of documented coronary artery disease prior to presentation nor an active cardiac condition, and given the emergent need for intervention further testing prior to this was not indicated; however we should keep in mind that the risk of serious cardiac complications increases significantly in these patients with multiple risk factors and advanced age, who are additionally undergoing an emergency procedure.

Current guidelines for preoperative cardiovascular evaluation do not recommend a preoperative 12-lead ECG or any other form of preprocedural cardiac assessment in asymptomatic patients undergoing low-risk interventions [1, 20], and endoscopy falls into a low-risk category according to the current classification. In this particular case, when patient presented to the Emergency Room he was completely asymptomatic and about to undergo an urgent low-risk intervention to remove the ingested foreign body, and the decision was to proceed with the endoscopic procedure given the urgency of his situation; however, we have to keep in mind that guidelines are exactly that, a guide, and not rigid rules. As in every decision in medicine, considerations regarding further preoperative evaluation have to be made according to the risk/benefit ratio of every individual patient, using available resources to estimate probabilities of effectiveness and adverse effects.

ECG is an inexpensive and very useful tool that could benefit some asymptomatic patients with multiple cardiovascular risk factors undergoing low-risk procedures and could be a cost-effective test to promote early diagnosis and treatment of cardiac complications. This case report summarizes an example of these adverse events aiming to increase awareness of this potentially fatal complication and highlight the relevance of prompt intervention and judicious use of preprocedural testing (e.g., ECG) despite guideline statements to the contrary. This underscores that guidelines are just that and are not rigid, unbreakable rules with proper documented justification.
